# Supplementary material for: Using core components in process evaluation: Passport skills for life
Source: PLoS One. 2026 Mar 31;21(3):e0346416. doi: 10.1371/journal.pone.0346416 (PMC13037957; doi:10.1371/journal.pone.0346416)
Supplement: S1 Table — (DOCX) [file pone.0346416.s001.docx]

**S1 Table. Implementation dimensions**

| **Dimension** | **Definition** |
| --- | --- |
| Fidelity (adherence) | The extent to which the program corresponds to the originally intended program |
| Dosage | Strength or quantity (in hours, sessions, etc.) Of the program |
| Quality | How well programme components are delivered by the programme facilitator |
| Participant responsiveness | The extent to which the program is engaging, interesting, and relevant to participants |
| Programme differentiation | The extent to which the programme differs from existing practices |
| Monitoring of control groups | Describing the nature and amount of the comparison conditions |
| Program reach | The rate of involvement and representativeness of program participants |
| Adaptation | Changes or modifications made to the original programme during implementation |

Adapted from: Durlak JA, DuPre EP. Implementation matters: A review of research on the influence of implementation on program outcomes and the factors affecting implementation. Am J Community Psychol. 2008;41(3-4):327-50. doi:10.1007/s10464-008-9165-0.
