# Supplementary material for: Using core components in process evaluation: Passport skills for life
Source: PLoS One. 2026 Mar 31;21(3):e0346416. doi: 10.1371/journal.pone.0346416 (PMC13037957; doi:10.1371/journal.pone.0346416)
Supplement: S2 Table — (DOCX) [file pone.0346416.s002.docx]

**S2 Table. FOI of Instructional Materials**

| **In Passport Skills for Life Curriculum and Materials**  Time   - Duration of unit - Time spent on instruction - Session Frequency   Order   - Investigation/lesson order - Order of Lesson Segments and Activities   Inclusion   - Inclusion of all essential segments within a lesson - Inclusion of all essential lessons   Pre-lesson   - Lesson overview - Lesson preparation   Essential program elements   - Materials presence - Writing structures - Readings - Assessments and assessment tools - Content of lesson - Procedures - Facts - Concepts - Processes - Use of class structures - Use of instructional delivery formats   Non-essential program elements   - Projects (PSL-specific) - Extensions (PSL-specific) - SEL-related - Not SEL related - Additional resources | **During Passport Skills for Life teacher implementation**  Facilitating student engagement with others   - Teacher facilitation of pair/group work - Teacher facilitation of student discussion   Facilitating student engagement with content   - Teacher facilitation of emotional literacy skills (in original work: Teacher facilitation of students doing potentially intellectually challenging work) - Teacher emphasis on types of content   Facilitating student role as learner   - Teacher facilitation of student autonomy - Teacher facilitation of student risk-taking - Teacher facilitation of student interest   Pedagogical strategies   - Teacher facilitation of use of materials, manipulatives, and tools - Teacher use of assessment to inform instruction. - Teacher use of differentiation - Teacher facilitation of multiple solution strategies (PSL specific) |
| --- | --- |

Adapted from: Century J, Rudnick M, Freeman C. A framework for measuring fidelity of implementation: A foundation for shared language and accumulation of knowledge. Am J Eval. 2010;31(2):199-218. doi:10.1177/1098214010366173.
