# Supplementary material for: Using core components in process evaluation: Passport skills for life
Source: PLoS One. 2026 Mar 31;21(3):e0346416. doi: 10.1371/journal.pone.0346416 (PMC13037957; doi:10.1371/journal.pone.0346416)
Supplement: S3 Table — (DOCX) [file pone.0346416.s003.docx]

**S3 Table. Standards for Reporting Qualitative Research (SRQR)**

| **No** | **Topic** | **Page** |
| --- | --- | --- |
|  | **Title and abstract** |  |
| S1 | Title | 1 |
| S2 | Abstract | 2 |
|  | **Introduction** |  |
| S3 | Problem formulation | 3 |
| S4 | Purpose or research question | 6 |
|  | **Methods** |  |
| S5 | Qualitative approach and research paradigm | 6 |
| S6 | Researcher characteristics and reflexivity | 6-7 |
| S7 | Context | 6-7 |
| S8 | Sampling strategy | 6-7 |
| S9 | Ethical issues pertaining to human subjects | 6 |
| S10 | Data collection methods | 7-8 |
| S11 | Data collection instruments and technologies | 8-9 |
| S12 | Units of study | 6-9 |
| S13 | Data processing | 8-9 |
| S14 | Data analysis | 8-9 |
| S15 | Techniques to enhance trustworthiness | 7-8-9 |
|  | **Results/findings** |  |
| S16 | Synthesis and interpretation | 9-19 |
| S17 | Links to empirical data | 9-19 |
|  | **Discussion** |  |
| S18 | Integration with prior work, implications, transferability, and contribution(s) to the field | 20-23 |
| S19 | Limitations | 23 |
|  | **Other** |  |
| S20 | Conflicts of interest | 24 |
| S21 | Funding | 24 |

Adapted from: O’Brien BC, Harris IB, Beckman TJ, Reed DA, Cook DA. Standards for reporting qualitative research: a synthesis of recommendations. Acad Med. 2014;89(9):1245-51. doi:10.1097/ACM.0000000000000388.
