# Supplementary material for: Using core components in process evaluation: Passport skills for life
Source: PLoS One. 2026 Mar 31;21(3):e0346416. doi: 10.1371/journal.pone.0346416 (PMC13037957; doi:10.1371/journal.pone.0346416)
Supplement: S4 Table — (DOCX) [file pone.0346416.s004.docx]

**S4 Table. The Content of the Passport Modules and Sessions**

| **Module** | **Topic of the session** | **Goal of the session** |
| --- | --- | --- |
| **Emotions** | 0. Beginning the adventure: the meeting | To stimulate children’s interest in participating in Passport programme activities. |
|  | 1. Valuing our differences and similarities | To help children identify, appreciate and value their differences and similarities. |
|  | 2. Understanding and expressing our emotions | To help children identify, name and talk about their own emotions, and to understand that we can sometimes feel different emotions at the same time. |
|  | 3. Recognising other people's feelings | To identify and then communicate our recognition of the emotions that someone else is feeling and to acknowledge that a person can feel more than one emotion at the same time, and that these emotions can sometimes be contradictory |
| **Relationships and helping each other** | 1. Helping each other and coping skills | To identify and evaluate coping skills which are focused on actions as well as those which are focused on emotions and to learn how to offer, ask for and accept help. |
|  | 2. Friendship | To explore how to make and keep friends. |
|  | 3. Challenges in friendship | To understand our reactions when we have to deal with rejection, abandonment and disappointment, and to identify coping strategies for dealing with these situations. |
| **Difficult situations** | 1. Dealing with frustration | To identify our reactions and coping skills in frustrating situations or in situations where we do not get our own way. |
|  | 2. Dealing with stress | To identify stress and how it feels inside, and to identify coping skills for dealing with it. |
|  | 3. Dealing with conflict | To identify and practise different coping skills in situations that involve conflict. |
| **Fairness, justice and what is right** | 1.Unfairness in daily life | To identify our feelings in unfair and unjust situations and to recognise our coping skills in these situations and to learn new ones. |
|  | 2. Dealing with bullying | To identify stress and how it feels inside, and to identify coping skills for dealing with it. |
|  | 3. Unfairness and injustice in the world | To identify our emotions when dealing with injustice and to find coping skills for dealing with these situations. |
| **Change and loss** | 1. Coping with change | To explore the consequences of change, our reactions to change, and to learn coping strategies for dealing with it. |
|  | 2. Coping with loss | To identify stress and how it feels inside, and to identify coping skills for dealing with it. |
|  | 3. Helping others in difficult situations | To identify our emotions when dealing with injustice and to find coping skills for dealing with these situations. |
|  | 4. Let's review – coping kit | To review everything we have learned in Passport. |
|  | 5. Celebration! | To celebrate what we have learned in Passport. |

Adapted from: Partnership for Children. Passport for 9–11 year olds [Internet]. London: Partnership for Children; [cited 2025 Jan 10]. Available from: https://www.partnershipforchildren.org.uk/what-we-do/programmes-for-schools/passport-for-life
