# Supplementary material for: Using core components in process evaluation: Passport skills for life
Source: PLoS One. 2026 Mar 31;21(3):e0346416. doi: 10.1371/journal.pone.0346416 (PMC13037957; doi:10.1371/journal.pone.0346416)
Supplement: Attachment — Submitted filename: review of article.docx [file pone.0346416.s005.docx]

**INTRODUCTION**

Discussion about implementation challenges is a bit repetitive. I think the program differentiation concept should be deeply unfolded. Please explain why a passport is theoretically interesting, and also add one statement on the explicit knowledge gap for a powerful introduction.

**Literature review**

There is dense layering of theoretical background, it can make the readers confuse specially with CC and FOI.

Frameworks should be supported with diagrams to make them more understandable.

Also, compare frameworks critically.

**Research questions**

RQ#3 is slightly general (What teacher behaviors were important?)

Please narrow it to observable indicators of implementation quality

**Methodology**

Samplinh's rationale should be elaborated further.

Please discuss transferability limits as well

**Intervention Description**

Overly descriptive in parts

Please include the visual program theory, and also clarify the expected pathways

**Data collection**

There is a limited explanation of document selection

Observations and interview

The duration of observation is not that clear

Please expand the justification of the short length

Also, clarify the reliability of observer training

**Data analysis**

Include a reflexivity statement

Please clarify the process of intercoder agreement

**Results**

There is a limited interpretation of CC's dominance

A strong link between teacher beliefs should be there

There should be clear evidence of selective implementation

Add comparative analysis between schools

Also, strengthen the link between student outcomes and quality indicators

**Table of evidence**

Tables are dense

Few categories overlap

Please simplify tables, and it is better to add interpretive summaries below each table

Link findings to universal design for learning also

**Discussion**

Please simplify theoartical language, and the conceptual model should be clear

**Limitations**

Some limitations should be elaborated, such as teacher selection bias, short duration of interviews, and cultural generalizability.

**Implications**

A few recommendations are broad; please specify them and discuss system-level training requirements

For future research student perspective is missing, which should be part of it.

Ethical transparency

AI acknowledgment is comparatively brief because requirements of some journals is detailed description
