# Supplementary material for: Using core components in process evaluation: Passport skills for life
Source: PLoS One. 2026 Mar 31;21(3):e0346416. doi: 10.1371/journal.pone.0346416 (PMC13037957; doi:10.1371/journal.pone.0346416)
Supplement: Attachment — Submitted filename: Response to reviewers.docx [file pone.0346416.s007.docx]

**THE JOURNAL’S Requirements**

Dear Editor,

Thank you for your careful review of our submission and for outlining the journal’s requirements. We have addressed each point as detailed below.

| 1.Please ensure that your manuscript meets PLOS ONE's style requirements, including those for file naming. | The manuscript files have been revised to comply with PLOS ONE style and file naming requirements. |
| --- | --- |
| 2. Please ensure that you include a title page within your main document. We do appreciate that you have a title page document uploaded as a separate file, however, as per our author guidelines we do require this to be part of the manuscript file itself and not uploaded separately. | A title page has now been included within the main manuscript file in accordance with the author guidelines. |
| 3. “Funding statement  This research was supported by a grant from the Kavli Trust Health Research Programme, funded by the Kavli Trust.  NH secured the funding. [[https://kavlifondet.no/en/category/projects-en/research-projects-en/programme-on-health-research-english/ [kavlifondet.no]](https://urldefense.com/v3/__https:/kavlifondet.no/en/category/projects-en/research-projects-en/programme-on-health-research-english/__;!!PDiH4ENfjr2_Jw!GpL-Iqmf4cvk8jvb_LaFg9q0Y4KPl-VAFwcuR81Osqn_CCQDQNbhsjoiqwSTmhAgKcwyGbiHAAluyaCs3Ic1i0cqpcY$)]  MA is externally funded by The Scientific and Technological Research Council of Turkey (TÜBİTAK) 2219 Program. [[https://tubitak.gov.tr/en [tubitak.gov.tr]](https://urldefense.com/v3/__https:/tubitak.gov.tr/en__;!!PDiH4ENfjr2_Jw!GpL-Iqmf4cvk8jvb_LaFg9q0Y4KPl-VAFwcuR81Osqn_CCQDQNbhsjoiqwSTmhAgKcwyGbiHAAluyaCs3Ic1E1uZmP4$)]  The views expressed in this paper are those of the authors and do not necessarily reflect the official policy or position of the Kavli Trust or TÜBİTAK.”  Please state what role the funders took in the study.  If the funders had no role, please state: "The funders had no role in study design, data collection and analysis, decision to publish, or preparation of the manuscript." | The following sentence has been added to the Funding Statement:  “The funders had no role in study design, data collection and analysis, decision to publish, or preparation of the manuscript.” |
| 4. Please note that funding information should not appear in any section or other areas of your manuscript. We will only publish funding information present in the Funding Statement section of the online submission form. Please remove any funding-related text from the manuscript. | All funding-related information has been removed from the main manuscript. |
| 5. We note that you have indicated that there are restrictions to data sharing for this study. PLOS only allows data to be available upon request if there are legal or ethical restrictions on sharing data publicly. For more information on unacceptable data access restrictions, please see [http://journals.plos.org/plosone/s/data-availability#loc-unacceptable-data-access-restrictions [journals.plos.org]](https://urldefense.com/v3/__http:/journals.plos.org/plosone/s/data-availability*loc-unacceptable-data-access-restrictions__;Iw!!PDiH4ENfjr2_Jw!GpL-Iqmf4cvk8jvb_LaFg9q0Y4KPl-VAFwcuR81Osqn_CCQDQNbhsjoiqwSTmhAgKcwyGbiHAAluyaCs3Ic1hMojDr8$).  Before we proceed with your manuscript, please address the following prompts:  a) If there are ethical or legal restrictions on sharing a de-identified data set, please explain them in detail (e.g., data contain potentially identifying or sensitive patient information, data are owned by a third-party organization, etc.) and who has imposed them (e.g., a Research Ethics Committee or Institutional Review Board, etc.). Please also provide contact information for a data access committee, ethics committee, or other institutional body to which data requests may be sent.  b) If there are no restrictions, please upload the minimal anonymized data set necessary to replicate your study findings to a stable, public repository and provide us with the relevant URLs, DOIs, or accession numbers. For a list of recommended repositories, please see  [https://journals.plos.org/plosone/s/recommended-repositories [journals.plos.org]](https://urldefense.com/v3/__https:/journals.plos.org/plosone/s/recommended-repositories__;!!PDiH4ENfjr2_Jw!GpL-Iqmf4cvk8jvb_LaFg9q0Y4KPl-VAFwcuR81Osqn_CCQDQNbhsjoiqwSTmhAgKcwyGbiHAAluyaCs3Ic13if5n2w$). You also have the option of uploading the data as Supporting Information files, but we would recommend depositing data directly to a data repository if possible.  We will update your Data Availability statement on your behalf to reflect the information you provide.  6. When completing the data availability statement of the submission form, you indicated that you will make your data available on acceptance. We strongly recommend all authors decide on a data sharing plan before acceptance, as the process can be lengthy and hold up publication timelines. Please note that, though access restrictions are acceptable now, your entire data will need to be made freely accessible if your manuscript is accepted for publication. This policy applies to all data except where public deposition would breach compliance with the protocol approved by your research ethics board. If you are unable to adhere to our open data policy, please kindly revise your statement to explain your reasoning and we will seek the editor's input on an exemption. Please be assured that, once you have provided your new statement, the assessment of your exemption will not hold up the peer review process.  7. In the online submission form, you indicated that your data is available only on request from a third party. Please note that your Data Availability Statement is currently missing [the name of the third party contact or institution / contact details for the third party, such as an email address or a link to where data requests can be made]. Please update your statement with the missing information. | "This study is based on qualitative observation and interview data involving detailed participant narratives. Although the data have been anonymised, they contain potentially identifiable and sensitive information relating to teachers. Consent for public data sharing beyond the research team was not obtained from participants, nor was such sharing approved by the University of Manchester Research Ethics Committee (Ref: 2022-14050-2440; original approval 30 June 2022; amendment approved 6 February 2024). Public deposition of the dataset would therefore breach the conditions of the approved ethics protocol.  The data are stored on secure, encrypted servers held by the University of Manchester.  All data access requests should be submitted to Professor Neil Humphrey for consideration. Access to available anonymised data may be granted through researcher collaboration with the University of Manchester. External researchers should contact the Office for Open Research ([openresearch@manchester.ac.uk](mailto:openresearch@manchester.ac.uk)).”  The Data Availability Statement in the submission system has been updated accordingly. |
| 8. Please ensure that you refer to Figure 1 in your text as, if accepted, production will need this reference to link the reader to the figure | Figure 1 is now explicitly referenced in the manuscript text. |
| 9. If the reviewer comments include a recommendation to cite specific previously published works, please review and evaluate these publications to determine whether they are relevant and should be cited. There is no requirement to cite these works unless the editor has indicated otherwise. | Thanks editor for the comment, we didnt receive any recommendations. |
| 10. Please review your reference list to ensure that it is complete and correct. If you have cited papers that have been retracted, please include the rationale for doing so in the manuscript text, or remove these references and replace them with relevant current references. Any changes to the reference list should be mentioned in the rebuttal letter that accompanies your revised manuscript. If you need to cite a retracted article, indicate the article’s retracted status in the References list and also include a citation and full reference for the retraction notice | The reference list has been reviewed for completeness and accuracy. No retracted articles are cited. |

**RESPONSE TO THE REVIEWERS**

We thank the reviewers for their thoughtful and constructive feedback. We have carefully addressed each comment and revised the manuscript accordingly. Below, we provide a point-by-point response.

**Reviewer 1**

| **Sampling clarity**  The manuscript reports observations across schools (numbers vary in sections four vs five schools mentioned). Please standardize and clarify the exact number of schools and selection criteria.  Clarify how observed lessons and interviewed teachers were selected (purposive, convenience, availability-based?). | Thank you for this comment. We have carefully reviewed the manuscript and standardised the number of participating schools throughout. The study included four mainstream primary schools.  Schools were selected using instrumental multiple case sampling to maximise contextual variation. Within each school, teachers delivering the intervention were invited to participate; therefore, teacher participation was based on role eligibility and availability rather than further purposive selection.  These clarifications have been added to the Design, context and participants section (pp.10-11). |
| --- | --- |
| Please clarify whether pilot observations were conducted; whether inter-coder or inter-observer agreement was assessed; how disagreements were resolved; number of coders involved in Phase 1 and Phase 2 coding; whether coding was double-coded; how the codebook evolved; and whether audit trails or memoing were used. | Thank you for this helpful request for clarification. We have expanded the Methods section to provide additional detail on your queries. Specifically:   - The interview schedule was pilot tested (as already noted in the manuscript); however, pilot observations were not conducted due to time constraints and school availability. - In Phase 1 (deductive content analysis of programme documents), coding was conducted by the first author. - In Phase 2 (reflexive thematic analysis), two authors independently coded approximately 20% of the dataset to develop and refine the coding framework. And the third author revised those. - Discrepancies were discussed and resolved through a consensus approach in regular analytic meetings. - The refined codebook was then applied to the full dataset by the first author. - In line with reflexive thematic analysis, no formal inter-coder reliability coefficient was calculated. - We also clarified that reflexivity was maintained throughout the analytic process via iterative codebook refinement, analytic annotations documented within NVivo, and regular team discussions in which assumptions were surfaced and interpretations critically examined.   These clarifications have now been added to the Data Analysis section (pp. 16-17). |
| Most conclusions are appropriately cautious; however, a few interpretive statements (e.g., mechanisms of erosion of treatment contrast) would benefit from more explicit linkage to specific observed patterns or counts already available in your tables. | Thank you for this helpful suggestion. We have strengthened the linkage between our interpretive statements and the empirical findings by explicitly referencing Table 2 in the Discussion section. We now clarify that our conclusions regarding erosion of treatment contrast are grounded in the documented patterns of omissions and adaptations observed across lessons.  See in the Discussion section (pp. 37-38). |
| Consider adding a short summary table mapping each identified core component to observed fidelity patterns. | Thank you for this constructive suggestion. We carefully considered adding a separate summary mapping table. However, given that Table 2 already presents the linkage between core components and observed fidelity patterns, we were concerned that an additional summary table would duplicate information. Instead, we revised Table 2 to make these linkages more explicit while maintaining coherence and readability across the manuscript. |
| A brief limitations subsection specifically addressing observer effect and programme familiarity would strengthen balance. | Thank you for this valuable suggestion. We agree that explicitly addressing potential observer effects and variation in programme familiarity strengthens the balance and transparency of the manuscript. We have revised the Strengths and Limitations section accordingly (pp.42-43). |
| If possible, add one short paragraph clarifying transferability beyond the regional context. | Thank you for this helpful suggestion. We have added a brief paragraph in Limitations clarifying the transferability of our findings beyond the Greater Manchester context. Specifically, we note that while Passport was originally developed in Canada and examined here in a UK setting, transferability to other cultural or policy environments should be considered in relation to contextual similarity and local implementation conditions. (p. 43 ) |

**Reviewer 2**

| **INTRODUCTION**  Discussion about implementation challenges is a bit repetitive. I think the program differentiation concept should be deeply unfolded. Please explain why a passport is theoretically interesting, and also add one statement on the explicit knowledge gap for a powerful introduction. | Thank you for this thoughtful and constructive feedback. We have revised the Introduction in several ways to strengthen the clarity:  First, we streamlined the discussion of implementation challenges to reduce repetition and improve coherence. Second, we clarified the theoretical distinctiveness of Passport by explicitly highlighting its grounding in coping and affect regulation theory, distinguishing it from SEL programmes that are atheoretical or aligned with a limited set of core theoretical frameworks. Third, we refined the articulation of the study’s knowledge gap by explicitly noting the limited research examining how practice and instructional core components interact with both fidelity and quality of delivery in real-world classroom settings.  These clarifications have been added to the (pp. 3-4). |
| --- | --- |
| **Literature review**  There is dense layering of theoretical background, it can make the readers confuse specially with CC and FOI.  Frameworks should be supported with diagrams to make them more understandable.  Also, compare frameworks critically. | Thank you for these helpful comments. To improve conceptual clarity and reduce potential layering, we simplified the description of the relationship between core components (CCs) and the Fidelity of Implementation (FOI) framework. Specifically, we streamlined the terminology and added a synthesising sentence clarifying that CC identification specifies the active ingredients of an intervention, whereas FOI provides a structure for assessing how those ingredients are delivered in practice (p. 7). In addition, we clarified the presentation of the FOI framework by explicitly directing readers to Supplementary Table S2 at its first mention in the text, where the framework is presented in detail (p. 7). |
| **Research questions**  RQ#3 is slightly general (What teacher behaviors were important?)  Please narrow it to observable indicators of implementation quality | We would like to thank the reviewer for suggestion, we reframed RQ3 as (p. 10);  What observable teacher behaviours and practices were associated with quality of delivery during the implementation of Passport? |
| **Methodology**  Please discuss transferability limits. | Thank you for this helpful suggestion. We have added a brief paragraph at the end of the Limitations clarifying the transferability of our findings beyond the Greater Manchester context. Specifically, we note that while Passport was originally developed in Canada and examined here in a UK setting, transferability to other cultural or policy environments should be considered in relation to contextual similarity and local implementation conditions. (p. 43 ) |
| **Intervention Description**  Overly descriptive in parts  Please include the visual program theory, and also clarify the expected pathways | We have revised the Intervention Description section. We integrated a theoretically grounded paragraph that makes explicit the hypothesised pathways of change. The revised text now clarifies how instructional components and structured rehearsal are intended to enhance coping flexibility, which in turn supports adaptive emotion regulation and disrupts maladaptive emotional cycles linked to internalising difficulties. We also explicate the expected social pathways, including strengthened peer connectedness and reductions in loneliness and bullying victimisation (p. 12).  Rather than introducing an additional figure, we elected to embed the programme theory directly into the narrative to maintain coherence. We believe this revision enhances conceptual clarity while preserving the focus of the manuscript. |
| **Data collection**  There is a limited explanation of document selection  Observations and interview  The duration of observation is not that clear  Please expand the justification of the short length | Thank you for these helpful comments. We have revised the Data Collection section (pp. 13-15).  First, we clarified that no selective sampling of documents was undertaken; rather, the full set of programme materials constituting the intended curriculum was systematically coded.  Second, we specified the scope and duration of classroom observations, clarifying that six teachers were each observed twice (twelve sessions in total), with each session lasting approximately 45–60 minutes.  Finally, we expanded the justification for the observational scope, noting that although the number of sessions was constrained by school scheduling and trial logistics, observing each teacher on two occasions enabled comparison. Observational data were also triangulated with document analysis and interviews to strengthen interpretive robustness. |
| **Data analysis**  Include a reflexivity statement  Please clarify the process of intercoder agreement | First, we have added a reflexivity statement consistent with reflexive thematic analysis, explicitly acknowledging how our theoretical positioning within SEL and familiarity with the Passport programme may have shaped analytic attention and coding decisions. We also clarified that reflexivity was maintained throughout the analytic process via iterative codebook refinement, analytic annotations documented within NVivo, and regular team discussions in which assumptions were surfaced and interpretations critically examined.  Second, we clarified the intercoder process. Approximately 20% of the dataset was independently coded by two authors. Coding discrepancies were discussed and resolved through consensus during analytic meetings, and the refined code ideas were subsequently applied to the full dataset. In line with the epistemological foundations of reflexive thematic analysis, no formal inter-coder reliability coefficient was calculated.  See pp. 15-17. |
| **Results**  A strong link between teacher beliefs should be there | Thank you for this comment. We would like to clarify that interpretation of CC dominance and its link to teacher beliefs was already embedded in both the Results and Discussion sections. Specifically, the Results highlight that teachers classified, prioritised, or omitted activities according to their perceived pedagogical importance and effectiveness, and interview excerpts explicitly illustrate how beliefs about classroom manageability and the limits of coping instruction shaped these decisions (p. 20). The Discussion further elaborates how method choice functioned as a proxy for pedagogical beliefs and how teachers’ emotional literacy and beliefs mediated the enactment of core components. To enhance clarity, we have made minor revisions to make this interpretive link more explicit (pp. 40-41). |
| There is a limited interpretation of CC's dominance | We respectfully note that the Results section explicitly identifies a structural dominance of lower-intensity, teacher-led formats over high-engagement components and links this pattern to teachers’ stated beliefs about effectiveness and classroom manageability. The implications of this dominance are then elaborated in the Discussion in relation to programme theory, treatment differentiation, and educator SEL. We therefore believe that the interpretation of CC dominance is substantively developed across the manuscript. |
| Add comparative analysis between schools  Also, strengthen the link between student outcomes and quality indicators | Thank you for this suggestion. While we acknowledge the potential value of cross-school comparison, the primary aim of the present study was to examine patterns of implementation in relation to core components rather than to conduct a comparative case analysis. Given the small number of participating schools and the process-focused design, we prioritised thematic depth over systematic between-school comparison. We have, however, clarified patterns of variation across schools where relevant in the Results section.  We also appreciate this suggestion regarding student outcomes. However, the present study was designed as a qualitative process evaluation focusing on fidelity and quality of implementation, rather than testing associations with student-level outcomes. While the broader trial examined programme effects, this analysis was not designed or powered to establish direct links between observed quality indicators and student outcomes.  **For complementary information, see;**   1. Humphrey N, Boehnke JR, Santos J, Alemdar M, Panayiotou M, O’Brien A, Burke L, Hamilton S, Qualter P, Demkowicz O, Lu Y, Thompson A. The effect of a universal, school-based social and emotional learning intervention (Passport: Skills for Life) on internalizing symptoms and related outcomes during the transition from childhood to adolescence: a cluster-randomized controlled trial. J Educ Psychol. 2025;117(7):1095-1114. doi:10.1037/edu0000963. 2. Demkowicz O, O’Brien A, Hamilton S, Burke L, Alemdar M, Mason C, et al. Child and school staff perceptions and experiences of universal social and emotional learning curricula in context: a qualitative case study registered report examining “Passport Skills for Life”. Br J Educ Psychol. 2026 Jan 30. |
| **Table of evidence**  Tables are dense  Few categories overlap  Please simplify tables, and it is better to add interpretive summaries below each table | Thank you for this thoughtful comment. We carefully considered whether and how the tables could be simplified. However, the apparent density and partial overlap between categories reflect the nature of the analytic process rather than issues of presentation. In reflexive thematic analysis, codes do not always fit into mutually exclusive or neatly bounded categories; some degree of conceptual overlap is expected and analytically meaningful.  We are cautious about removing overlap or collapsing categories, as doing so would risk oversimplifying the findings and misrepresenting the interpretive depth of the analysis. Our intention in retaining this structure is to preserve transparency regarding how themes and sub-themes relate to one another. |
| Link findings to universal design for learning also | Thank you for this insightful suggestion. We agree that the principles of Universal Design for Learning (UDL) offer a useful lens for interpreting implementation quality, particularly in relation to engagement, multimodal instruction, and equitable access.  In response, we have integrated a brief conceptual link within the Discussion section, as the manuscript already has theoretical density, specifically in the section (see p. 39) addressing instructional methods and differentiated delivery. We highlight how the observed reduction of high-engagement formats (e.g., role-plays, games) to lower-intensity whole-class discussion narrowed the programme’s multimodal design and limited opportunities for embodied rehearsal and inclusive participation—elements aligned with UDL’s emphasis on multiple means of engagement and representation. |
| **Discussion**  Please simplify theoretical language, and the conceptual model should be clear | Thank you for this helpful observation. We have revised the Discussion to improve conceptual clarity and reduce theoretical density. Specifically, we revised the opening section of the Discussion to make the underlying conceptual pathway more explicit by clearly articulating how SEL impact is theorised to depend on the enactment of core components (CCs), how enactment varies in intensity and depth, how practice and instructional CCs operate interdependently, and how instructional mechanisms (e.g., engagement, responsiveness, use of materials, differentiation) mediate their activation within the classroom.  See p. 34. |
| **Limitations**  Some limitations should be elaborated, such as teacher selection bias, short duration of interviews, and cultural generalizability. | Thank you for this helpful suggestion. We have expanded the *Strengths and Limitations* section to address these points more explicitly (pp. 42-43).  Specifically, we now:   - Acknowledge the possibility of **teacher selection bias**, noting that participation in the qualitative component may reflect self-selection and differences in confidence or engagement with the programme. - Clarify the rationale for the **short duration of interviews** (approximately 15 minutes), explaining that this design choice was made to minimise burden in busy school contexts while recognising that it limited depth of exploration. - Elaborate on issues of **cultural transferability**, emphasising that Passport was developed in Canada and implemented in English primary schools, and that transferability to other cultural and policy contexts should be considered cautiously in light of contextual variability. |
| **Implications**  For future research student perspective is missing, which should be part of it. | Thank you for this valuable suggestion. We agree that integrating student perspectives can strengthen understanding of implementation processes. While student perspectives were explored in complementary qualitative work arising from the same broader trial [7], we have now clarified in the Implications section that future studies could more explicitly integrate teacher and student perspectives within a single analytic framework (see p. 45). |
| Ethical transparency | Ethical procedures, including institutional approval, informed consent, and anonymity safeguards, are detailed in the Methods section (p. 11). |
| AI acknowledgment is comparatively brief because requirements of some journals is detailed description | We have expanded the AI acknowledgment to provide greater transparency. We now clarify that AI tools (ChatGPT-4.0 and DeepL) were used solely for language editing (grammar, spelling, and fluency), that no analytic or interpretive tasks were conducted using AI, and that all intellectual content, data analysis, and interpretations remain the responsibility of the authors.  See p.46 |
